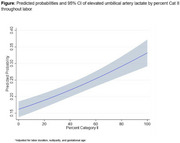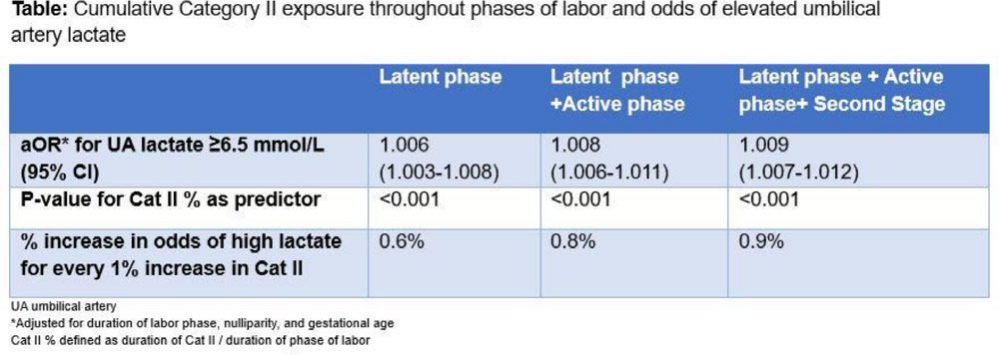# Oral Concurrent Session 5 – Clinical Obstetrics: Thursday, February 12, 2026 1:00 PM – 3:30 PM

**DOI:** 10.1002/pmf2.70159

**Published:** 2026-02-09

**1:00 PM – 1:15 PM**

## 54 | Adoption and Real‐World Effects of Azithromycin for Labored Cesarean Birth on Postpartum Infections

Taylor S. Freret^1^; Ethan Litman^2^; Mark A. Clapp^3^; Sarah E. Little^4^

^1^Beth Israel Deaconess Medical Center, Brookline, MA; ^2^Beth Israel Deaconess Medical Center, Boston, MA; ^3^Department of Obstetrics and Gynecology, Mass General Brigham, Massachusetts General Hospital, MA; ^4^Beth Israel Deaconess Medical Center, Newton, MA

**Objective**: The C/SOAP trial (late 2016) showed that perioperative azithromycin for cesarean birth after labor reduces postpartum infection. We sought to analyze the uptake and effect of azithromycin on postpartum infection in a nationwide cohort outside of a clinical trial.

**Study Design**: This was a difference‐in‐differences (DID) analysis (2013–2024) using the nationwide Epic Cosmos platform. Laboring persons without IAI who gave birth to a liveborn singleton at 24–43 weeks were included. The primary exposure was perioperative azithromycin. In the DID design, cesarean births (treatment group) were compared to vaginal births (control group); the pre‐period was 2013–2016, and the post‐period 2017–2024. The primary outcome, adapted from the C/SOAP trial, was a composite of endometritis, wound infection, or other infection within 6 weeks, identified by ICD codes. The DID analysis used patient‐level linear regression, adjusting for age, parity, delivery BMI, diabetes, prior cesarean birth, gestational age, ROM ≥ 18 hours, and reaching the second stage.

**Results**: The cohort included 1,636,152 individuals; 212,030 (13.0%) delivered by cesarean. Azithromycin was administered to 73,969 (34.9%) of the cesarean and 442 (0.03%) of the vaginal births. There was a rapid increase in use among cesarean births after 2016, from 2.1% in 2013 to 61.2% in 2024 (Figure 1). There was no increase in use among vaginal deliveries. Among cesarean births, 6.8% who received azithromycin and 8.0% of those who did not developed an infectious complication (*p* < 0.001). The rate of infection increased from 1.9% to 2.6% among vaginal births and decreased from 8.6% to 7.4% among cesarean births between the two periods (Figure 2). In the adjusted DID analysis, azithromycin reduced the incidence of infectious complications by −2.1 [−2.3, −1.9] percentage points (∼22% reduction).

**Conclusion**: In a nationwide cohort, there was rapid uptake of azithromycin administration after cesarean birth, which decreased postpartum infectious complications. These findings demonstrate external validity of the C/SOAP trial findings in contemporary obstetric practice.

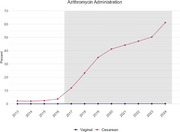

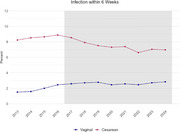

**1:15 PM – 1:30 PM**

## 55 | **Intravenous Papaverine Prior to Dinoprostone for Labor Induction: A Randomized Double‐Blinded Placebo‐Controlled Trial**

Raneen Abu Shqara^1^; Yara Bishara^2^; Nadir Ganem^3^; Inshirah Sgayer^2^; Miri Lavinsky^4^; Lior Lowenstein^3^; Maya Frank Wolf^3^

^1^Galilee Medical Center, Nahariya, HaZafon; ^2^Galilee Medical Center, Nahariyya, HaZafon; ^3^Galilee Medical Center, Naharyia, HaZafon; ^4^Galilee Medical Center, Galilee Medical Center, HaZafon

**Objective**: Papaverine has been hypothesized to facilitate cervical change during labor induction. We evaluated Bishop scores and the induction‐to‐delivery interval, after intravenous papaverine administration prior to dinoprostone insertion.

**Study Design**: In a double‐blinded trial, we randomized term parous patients with singleton gestations and a Bishop score ≤6 to receive 80 mg of intravenous papaverine in 100 mL of 0.9% saline or 100 mL of 0.9% saline (placebo), within 30 minutes before vaginal insertion of dinoprostone. The primary outcomes were: a change in Bishop score from dinoprostone insertion to removal, and the induction‐to‐delivery interval.

**Results**: For the papaverine (n = 55) compared to the placebo group (*n* = 55), the median change in Bishop score was higher (4.6 vs. 3.5; *p* = .015) and the median induction‐to‐delivery interval was shorter (15.6 vs. 21.7 hours; *p* = .030). For the papaverine group, the interval from dinoprostone insertion to removal was shorter (11.7 vs. 16.1 hours; *p* = .030) and spontaneous rupture of membranes occurred more frequently (60% vs. 40%; *p* = .036). A greater proportion of women in the papaverine group achieved a Bishop score >8 (69.1% vs. 49.1%; *p* = .032). Cesarean delivery rates were similar between the groups. The proportions of patients in the papaverine and placebo groups that required an additional cervical ripening method did not differ statistically (*p* = .142). Statistically significant differences were not observed in maternal pain scores, satisfaction, or neonatal outcomes. Papaverine administration predicted a shorter induction‐to‐delivery interval (*B* = –8.46 hours, *Standard Error* = 3.42, *p* = .015), after adjusting for maternal age, body mass index, parity, gestational age at induction, and baseline Bishop score.

**Conclusion**: Intravenous papaverine versus a placebo, administered prior to dinoprostone vaginal insertion in parous patients, resulted in a greater change in Bishop score and a shortened induction‐to‐delivery interval. Papaverine may serve as a beneficial adjunct to dinoprostone vaginal insertion.

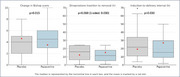

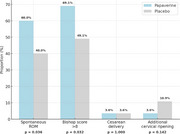

**1:30 PM – 1:45 PM**

## 56 | **Effect of Oral Calcium Carbonate on Uterine Contractility and Labor Outcomes: A Randomized Controlled Trial**

Tina M. Bui; Michelle Nelson; Preeanka Mazumder; Rabia Rehman; Kristina A. Roloff; Guillermo Valenzuela

Arrowhead Regional Medical Center, Colton, CA

**Objective**: Labor dystocia is a leading indication for cesarean delivery in the United States and may, in part, result from ineffective contractions. Calcium plays an essential role in myometrial contractility. In this study, we hypothesized that oral calcium supplementation may improve uterine responsiveness and aimed to evaluate the effect of oral calcium carbonate on uterine activity.

**Study Design**: We conducted a randomized controlled trial at a single tertiary care hospital. Term patients with singleton pregnancies and an intrauterine pressure catheter were randomized to receive 2,000 mg of oral calcium carbonate or no intervention. Exclusion criteria included prior cesarean delivery, contraindications to calcium supplementation, or use of medications affecting uterine tone (e.g., magnesium sulfate, terbutaline, or misoprostol within four hours).

Uterine activity was measured using Montevideo units (MVUs), contraction frequency, and peak pressure at baseline and every 30 minutes for two hours following the intervention. Oxytocin dosing was held constant during the study period. Outcomes were compared using two‐tailed t‐tests.

**Results**: Eighty‐nine patients were included in the analysis (45 control, 44 intervention). Baseline demographic characteristics and uterine activity were similar between groups. At 30 minutes post‐intervention, the calcium group demonstrated significantly higher MVUs compared to controls (171.9 ± 61.9 vs. 145.1 ± 64.9 mmHg, *p* = 0.05). No significant differences in MVUs were observed at 60, 90, or 120 minutes. Peak contraction pressure, contraction frequency, oxytocin dose, estimated blood loss, and labor duration were similar between groups.

**Conclusion**: Oral calcium carbonate was associated with a transient increase in uterine contractility at 30 minutes. Further studies are needed to determine whether higher or repeated dosing could have a clinically meaningful impact on uterine contractility or labor duration.

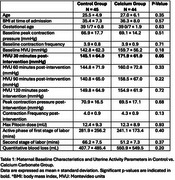

**1:45 PM – 2:00 PM**

## 57 | **Azithromycin Dosing in Preterm Premature Rupture of Membranes (PPROM): Population Pharmacokinetics and Dosing Regimen Optimization**

Rupsa C. Boelig^1^; Kevin Lam^2^; Viren Soni^2^; Ankit Rochani^3^; Gagan Kaushal^4^; Amanda Roman^2^; Walter Kraft^2^

^1^Sidney Kimmel Medical College, Thomas Jefferson University, Philadelphia, PA; ^2^Thomas Jefferson University, Philadelphia, PA; ^3^St. John Fisher University, Rochester, NY; ^4^Thomas Jefferson University, Thomas Jefferson University, PA

**Objective**: Compare amniotic fluid pharmacokinetic (PK) parameters of 1g once vs 500 mg daily dosing of azithromycin for PPROM and simulate dosing to identify the optimal regimen that maintains amniotic fluid azithromycin concentration over the minimum inhibitory concentration (MIC) of common genitourinary (GU) pathogens found with intraamniotic infection.

**Study Design**: This is a prospective study of singletons with PPROM who received 1g once or 500 mg dailyx7d of azithromycin at two sites per respective site protocol. Maternal plasma was collected pre‐dose, 1–4 and 12–24 hours post‐dose, and every 24 hours thereafter. Amniotic fluid was collected opportunistically non‐invasively by extracting amniotic fluid from sanitary pads. Population PK analysis performed with Monolix version 2024R1. Multiple oral dosing regimens simulated to estimate amniotic fluid exposure over a seven‐day period. Data presented as median and interquartile range (IQR).

**Results**: 18 participants with 101 plasma and 223 amniotic fluid samples were included. A two‐compartment model with first‐order absorption best described the plasma data. Azithromycin exposure in the first 24 hours was greater with 1 g once (AUC_0‐24_/MIC 27.84 (9.01, 71.77)  vs 13.84 (4.52, 36.44), *p* < 0.01). Azithromycin concentration by day 7 (27.46 (10.42, 97.85) vs 5.92 (2.07, 21.86) ng/ml, *p* < 0.01) as well as time over minimum inhibitory concentration of common genitourinary pathogens, even at the lowest desired concentration of >20 ng/ml (86.14 (27.87, 98.23) vs 64.66 (0, 99.28) hrs, *p* < 0.01) were greater with daily dosing compared to 1 g once. Simulated dosing regimens suggest that a loading dose followed by daily (1 g once then 500 mg daily for six days) or alternate day (2g once, 1 g days 2 and 4) dosing most rapidly and consistently maintain amniotic azithromycin concentration >60 ng/ml over a seven‐day period (Figure).

**Conclusion**: 500mg azithromycin dailyx7 days is superior to 1g once at maintaining amniotic fluid azithromycin concentrations over the MIC of common GU pathogens. The optimal simulated dosing regimen is a loading dose followed by daily or alternate day dosing.

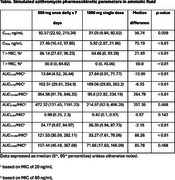

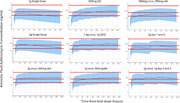

**2:00 PM – 2:15 PM**

## 58 | **Non‐Inferiority Analysis of an FHR AI Algorithm to Clinician Interpretation**

Rohit Pardasani^1^; Karen P. Becker^2^; Renee Vitullo^1^; Sara M. Harris^3^; Jaydev Kamani^4^; Jack Page^4^; Kritika Garg^4^; John Beard^1^

^1^GE HealthCare, Waukesha, WI; ^2^GE HealthCare, waukesha, MD; ^3^GE HealthCare, Cape Coral, FL 33991‐3441, FL; ^4^GE HealthCare, waukesha, WI

**Objective**: Cardiotocography (CTG) is used in obstetric care for monitoring fetal heart rate (FHR) and uterine activity (UA) during labor. Clinician interpretation of CTG tracings may be subjective with inter‐ and intra‐observer variability. An Artificial Intelligence (AI) algorithm prototype using deep learning and rule‐based techniques was developed to label FHR and UA data to support clinician interpretation. This study evaluated the non‐inferiority of the prototype algorithm's performance compared to clinical reader interpretations.

**Study Design**: This retrospective non‐inferiority evaluation utilized a healthcare dataset which included CTG data at five US hospital facilities between 2013–2024. The interpretations by the prototype AI algorithm were compared to clinical readers (Figure 1) on primary (baseline value, accelerations, decelerations, contraction frequency, and contraction duration) and secondary endpoints (variability and deceleration types), through a panel of qualified expert clinicians who accepted or rejected the interpretations. A maximum threshold of 15% was required between the clinical reader and the algorithm to establish non‐inferiority.

**Results**: Of the 2639 de‐identified fetal tracings, 800 were randomly selected for this study. The prototype algorithm showed comparable results to the clinical reader for all endpoints (Figure 2), with percent differences (95% CI) in acceptance rates (AR) of 3.38% (−1.5%, 8.25%) for baseline value, 3.03% (0.72%, 5.34%) for acceleration, 7.14% (3.68%, 10.61%) for deceleration, 8.25% (3.57%, 12.93%) for contraction frequency, 22.13% (17.39%, 26.86%) for contraction duration, −1.75% (−4.10%, 0.60%) for variability, and 9.13% (5.38%, 12.88%) for deceleration type. The AR for contraction duration decreased to 13.75% when the output was modeled for a 20‐second range of variability.

**Conclusion**: This evaluation demonstrated the non‐inferiority of a AI algorithm prototype to analyze and interpret fetal tracings when compared to clinical readers. These findings highlight AI's potential to support decision‐making in obstetric care.

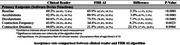

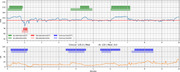

**2:15 PM – 2:30 PM**

## 59 | **Second Stage Cesarean Delivery: A Mixed‐Methods Exploration of Techniques and Uncertainty Among U.S. Clinicians**

Janayah McClellan^1^; Erica Marion^1^; Sarah T. McMahon^1^; Melissa A. Clark^2^; Anna Palatnik^3^; Brock E. Polnaszek^1^

^1^Medical College of Wisconsin, Milwaukee, WI; ^2^Brown University School of Public Health, Providence, RI; ^3^Medical College of Wisconsin, MILWAUKEE, WI

**Objective**: Second stage cesarean delivery is a potential emergency associated with maternal and neonatal morbidity, yet no consensus exists on optimal management. We aimed to explore techniques used and uncertainty perceived by clinicians during management of second stage cesarean delivery.

**Study Design**: A mixed methods study was conducted from January 2025 to April 2025 using a convenience sample of clinicians performing second stage cesarean delivery in the United States. We collected quantitative data from surveys and qualitative data from semi‐structured individual interviews. The surveys assessed clinicians’ perceptions of second stage cesarean techniques, management, uncertainty, evidence gaps, and impact on maternal and/or neonatal morbidity.

**Results**: Among clinicians (*n* = 406 surveys; *n* = 41 interviews), wide variation in technique was reported across providers and delivery settings (Table 1). The five techniques perceived as safest in descending order included fetal pillow (38.9%), push or hand from below (38.4%), reverse breech extraction (37.1%), head de‐stationing (36.2%), dorsal lithotomy positioning (27.3%). Perception of technique varied significantly based on the verbs (e.g., harm, reduce, benefit, data to support) used to assess the delivery technique's impact on morbidity. Most clinicians perceived that the technique more frequently influenced maternal rather than neonatal morbidity, while some acknowledge not knowing the impact on neonatal morbidity. Two major themes emerged from interviews: 1) Insufficient data limits consensus on optimal technique, and 2) Clinicians often perceive maternal and neonatal morbidity as competing priorities (Table 2).

**Conclusion**: Clinicians face significant uncertainty balancing maternal and neonatal morbidity during second stage cesarean delivery. The lack of comparative effectiveness data on delivery technique limits standardization and evidence‐based practice. Prospective studies are urgently needed to compare techniques and inform clinical guidelines.

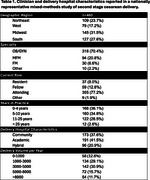

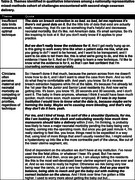

**2:30 PM – 2:45 PM**

## 60 | **Membrane Sweeping Versus Double‐Balloon Catheter for Cervical Ripening: A Randomized Controlled Trial**

Renana Izakson^1^; May Weinberg^2^; Michal Kovo^3^; Tal Biron‐Shental^4^; Gal Cohen^4^

^1^Meir Medical Center, meir medical center, HaMerkaz; ^2^Meir Medical Center, kfar saba, HaMerkaz; ^3^Shamir Medical Center, Beer Yaacov, HaMerkaz; ^4^Meir Medical Center, Kfar Saba, HaMerkaz

**Objective**: Membrane sweeping (MS) is a simple, safe, and cost‐effective method to promote spontaneous labor at term. Compared to no intervention, MS reduces the need for induction and is as effective as prostaglandins or oxytocin in achieving vaginal delivery. We aimed to compare the effectiveness of MS vs. double‐balloon catheter (DBC) for cervical ripening in term pregnancies.

**Study Design**: A prospective randomized controlled trial at a tertiary center (2024–2025) including term pregnancies requiring cervical ripening, with a cervix suitable for either MS or DBC. Exclusion criteria were prior ripening or cesarean delivery. After consent, participants were randomized to MS (performed twice: at admission and 4–6 hours later) or DBC (inserted for 12 hours). The primary outcome was Δ Bishop score before and 12 hours post‐intervention. Secondary outcomes included time to cervical ripening and delivery, oxytocin use, maternal/neonatal outcomes, pain (VAS) and satisfaction. Sample size powered for non‐inferiority in Δ Bishop score required ≥48 participants per group. Analyses included intention‐to‐treat and per‐protocol.

**Results**: Of 114 women randomized (MS:60; DBC:54), 107 were analyzed per protocol (MS:57; DBC:50). Groups had similar baseline characteristics and Bishop scores. At 12 hours, median Δ Bishop scores were comparable (2 [1–3] vs. 2 [1–4], *p* = 0.212). Ripening duration, VAS and satisfaction scores were also similar. MS was associated with lower oxytocin use (63.8% vs. 94.3%, *p* < 0.001) and shorter delivery room stay (8.9 + 6.0h vs. 12.8 + 7.8h, *p* = 0.002). In per‐protocol analysis, delivery within 16 hours was more frequent with MS (35.1% vs. 16.0%, *p* = 0.025), with a similar trend at 20 hours (45.6% vs. 28% *p* = 0.06). No differences were observed in tachysystole, delivery within 24 hours, mode of delivery, or obstetric outcomes.

**Conclusion**: MS was non‐inferior to DBC in Δ Bishop score, with the additional advantages of reduced oxytocin use and shorter time to delivery—possibly due to its physiological effect. As a minimally invasive option, MS may be preferable for selected term pregnancies requiring cervical ripening.

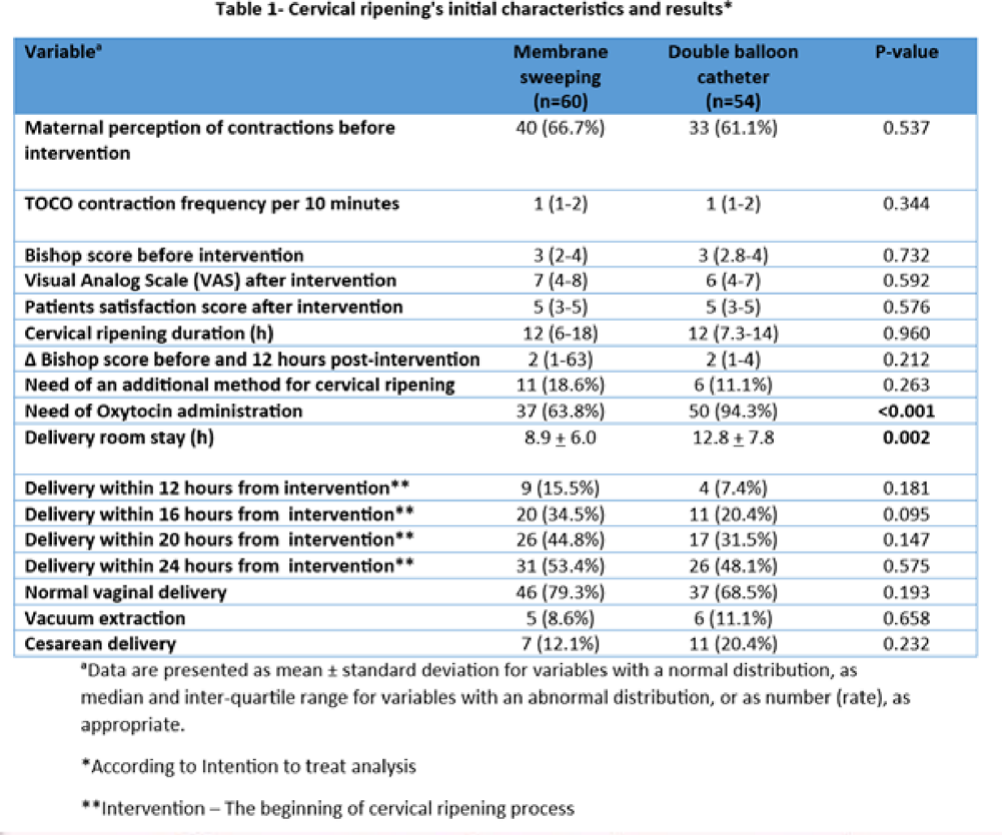

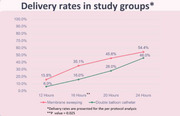

**2:45 PM – 3:00 PM**

## 61 | **Factors Associated With a Restart of Oxytocin After Discontinuation in Active First Stage of Labor**

Charles Garabedian^1^; Loïc Sentilhes^2^; Raoul Desbrière^3^; Paul Berveiller^4^; Diane Korb^5^; Charline Bertholdt^6^; Julie Carrara^7^; Norbert Winer^8^; Camille Le Ray^9^; Aude Girault^9^

^1^CHU Lille, CHU Lille, Department of obstetrics, Univ. Lille, ULR2694 – METRICS, F59000 Lille, France, Nord‐Pas‐de‐Calais; ^2^Bordeaux University Hospital, Bordeaux Universitary Hospital, Aquitaine; ^3^Department of Obstetrics and Gynecology, Hôpital Saint Joseph, Marseille, France, Marseille, Midi‐Pyrenees; ^4^Department of Obstetrics and Gynecology, Centre Hospitalier Intercommunal de Poissy/ Saint‐Germain‐en‐Laye, Rue du Champ Gaillard, Poissy Cedex, France, Poissy, Ile‐de‐France; ^5^Department of Obstetrics and Gynecology, Robert Debré Hospital, AP‐HP, Paris, France, Paris, Ile‐de‐France; ^6^University of Lorraine, CHRU NANCY, Obstetrics and Gynecology Department, NANCY, France, Nancy, Lorraine; ^7^Department of Obstetrics and Gynecology, Antoine Béclère Hospital, AP‐HP, Paris, France, Clamart, Ile‐de‐France; ^8^Department of Obstetrics and Gynecology, University Hospital of Nantes, Nantes, Pays de la Loire; ^9^Department of Obstetrics and Gynecology, Port‐Royal Maternity Hospital, AP‐HP, Cochin Hospital, FHU PREMA, Paris, France, Paris, Ile‐de‐France

**Objective**: In trials on oxytocin discontinuation during active labor, 30–40% of women required its restart. Identifying factors associated with restart could help guide individualized care. This study aimed at identifying the factors associated with the restart of oxytocin after its discontinuation in active labor.

**Study Design**: This is a secondary analysis of a multicenter randomized controlled trial conducted across 21 maternity centers in France between January 2020 and January 2022. Participants were divided into two groups: the oxytocin restart group and the oxytocin discontinuation group. A multivariable multilevel logistic regression model with random intercepts for centers was used to identify factors associated with oxytocin restart.

**Results**: Of the 833 women included, 367(44.1%) had oxytocin restarted and 466 (55.9%) delivered without oxytocin resumption. The rate of nulliparous was significantly higher in the restart group compared to the discontinuation group (72.2% vs 37.6%, *p* < .01). Oxytocin was restarted in 60.2%(265/440) of nulliparous women and 25.9%(102/393) of parous women, *p* < .01. Cervical ripening was more frequent in the restart group than in the discontinuous group (55.6% vs 38.4%, *p* < .01). The median time from oxytocin onset to active labor was longer in the restart group (295 min, IQR 197–442) than in the discontinuation group (233 min, IQR 160–329, *p* < .01), with a delay exceeding 6 hours observed more often in the restart group (36.0% vs 20.4%, *p* < .01). Median cervical dilation at oxytocin discontinuation was lower in the restart group (7 cm, (6–9)) compared to the discontinuation group (8 cm, (6–10)), *p* < .01. Multivariable multilevel analysis identified two independent factors associated with oxytocin resumption: nulliparity aOR 4.10, 95% CI [2.89–5.82] and a time interval >6 hours between oxytocin onset and active labor aOR 1.72, 95% CI [1.19–2.50].

**Conclusion**: In this secondary analysis of a large randomized controlled trial, nulliparity and slow labor progression were independently associated with oxytocin restart after its discontinuation during the active first stage of labor.

**3:00 PM – 3:15 PM**

## 62 | **Prelabor Maternal Pushing Training Using Visual Biofeedback via Self‐Operated Home Ultrasound: A Randomized Controlled Study**

Natav Hendin^1^; Amit Shachak^2^; Dorit Asher^3^; Eran Hadar^3^; Yael Benyamini^4^; Sharon Perlman^3^

^1^Rabin Medical Center, Petah Tikva, HaMerkaz; ^2^Rabin medical center, Petach Tikva, HaMerkaz; ^3^Rabin Medical Center, Petach Tikva, HaMerkaz; ^4^Tel Aviv University, Tel Aviv, Tel Aviv

**Objective**: This randomized controlled study evaluates whether pre‐labor visual biofeedback, using a self‐operated home ultrasound device, can improve maternal labor outcomes.

**Study Design**: Nulliparous women at 37–41 weeks’ gestation planning vaginal delivery were randomized into three groups: Group 1 received routine obstetric ultrasound + biofeedback guided by an obstetrician. Group 2 received the same, plus a home ultrasound device for practice. The control group underwent routine ultrasound without biofeedback. The self‐operated ultrasound device, connected to a smartphone, provided real‐time visual biofeedback of fetal head descent via angle of progression (AoP). Participants in the biofeedback groups were taught to recognize landmarks and visualize AoP during coached pushing. Pushing efforts were recorded, and participants completed psychological assessments pre‐ and post‐intervention.

The Primary outcome was second stage duration, with secondary outcomes including psychological characteristics, mode of delivery, perineal tears, and neonatal complications.

**Results**: Ninety participants were randomized; 71 were analyzed after exclusions. The mean number of home training sessions was 3.5 per participant, with all enabling AoP and ∆AoP assessment. The mean ∆AoP during home training was 14.7°.

When comparing maternal outcomes between the groups, the shortest second‐stage labor duration was observed in the hospital + home biofeedback group (58 minutes). The hospital‐only biofeedback group and the control group had a second‐stage duration of 124 and 103 minutes respectively.

The proportion of vacuum‐assisted deliveries was lowest in the home biofeedback group (2/19, 10%) compared to the hospital‐only biofeedback group (6/24%, 52) and the control group (19%5/27,). Participants in the home ultrasound group reported feeling more in control, less anxious, and better prepared for labor.

**Conclusion**: These preliminary findings suggest that self‐operated home ultrasound for pushing training is feasible, acceptable, and may improve labor outcomes by shortening the second stage and reducing operative delivery.

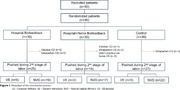

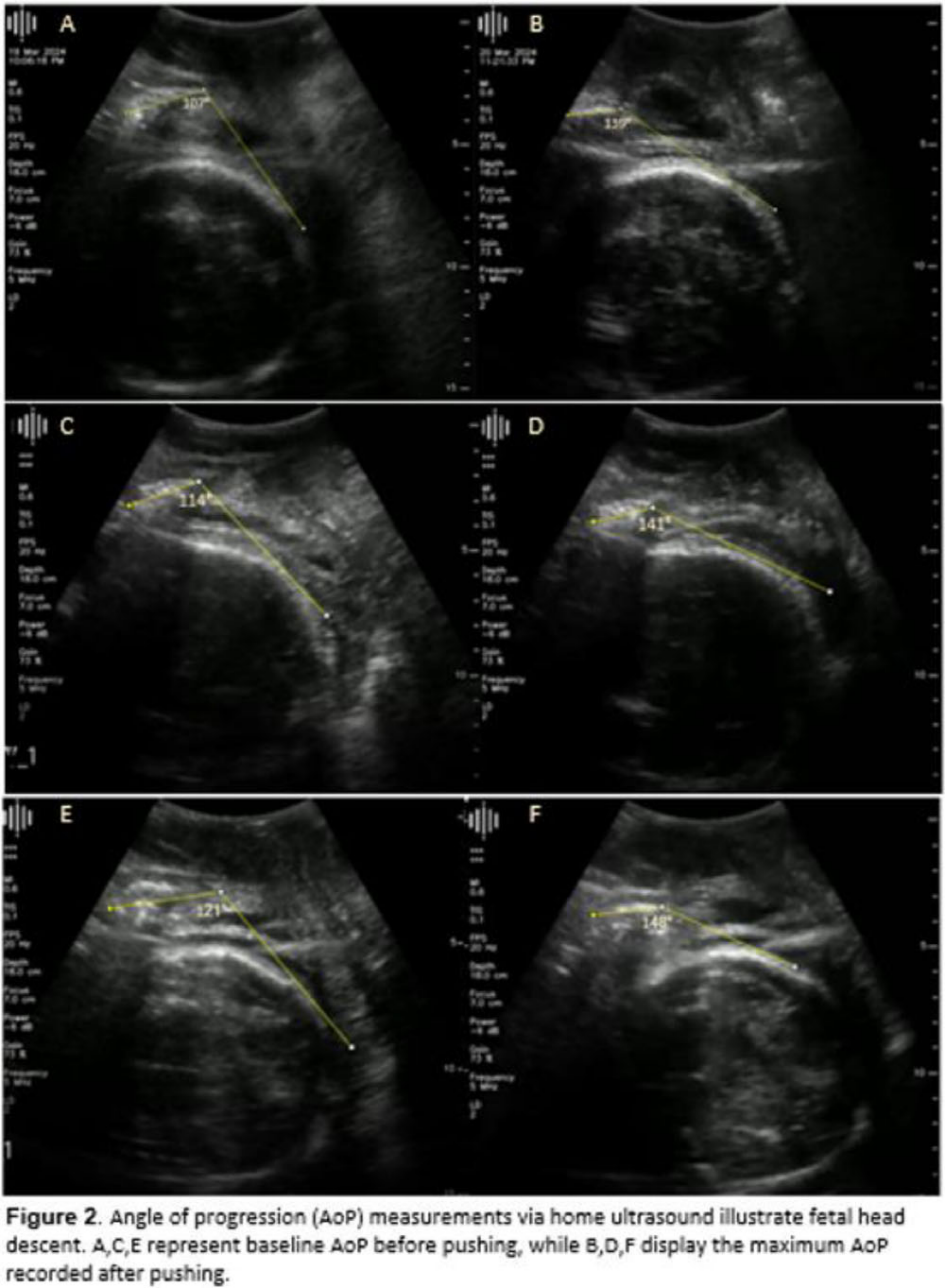

**3:15 PM – 3:30 PM**

## 63 | **The Cumulative Impact of Category II Tracings Throughout Labor on Neonatal Lactate**

Nandini Raghuraman^1^; Antonina I. Frolova^2^; Megan L. Lawlor^2^; Amanda C. Zofkie^2^; Roxane Rampersad^2^; Jeannie C. Kelly^1^; Steve Porter^3^; Susan Mann^4^; George A. A. Macones^5^; Alison G. G. Cahill^6^

^1^Washington University School of Medicine, Washington University School of Medicine, MO; ^2^Washington University School of Medicine, St. Louis, MO; ^3^Case Western Reserve University, University Hospitals MacDonald Women's Hospital, Cleveland, OH; ^4^Beth Israel Deaconess Medical Center, Harvard Medical School, Boston, MA; ^5^Department of Women's Health, Dell Medical School at the University of Texas at Austin, Austin, TX; ^6^Department of Women's Health, Dell Medical School at the University of Texas at Austin, Department of Women's Health, Dell Medical School at the University of Texas at Austin, TX

**Objective**: Although Category II (Cat II) fetal heart rate (FHR) tracings are considered indeterminate for predicting acidemia, little is known about risks of Cat II accumulation over time. We evaluated the association between the proportion of labor in Cat II and the risk of elevated umbilical artery (UA) lactate, a marker of fetal hypoxia and metabolic acidosis.

**Study Design**: This retrospective cohort included all laboring patients from 3/2023–2/2025 with continuous FHR monitoring on a single unit with universal cord gases. FHR category was documented every 30 minutes in the EHR by bedside nurses and extracted via an integrated clinical decision support tool. The primary exposure was percent time in Cat II (Cat II %), defined as the duration of Cat II over the duration of labor, calculated overall and by labor phase. The outcome was elevated UA lactate (≥6.5 mmol/L). Multivariable logistic regression was used to estimate adjusted odds ratios (aORs) and predicted probabilities, adjusting for labor duration, nulliparity, and preterm birth. Stepwise models assessed cumulative associations across labor phases.

**Results**: Among 4191 patients with EFM documentation and cord gases, 600 (14.3%) had an elevated UA lactate. Mean Cat II % was 42±33%. Increasing Cat II % was significantly associated with elevated UA lactate (*p* < 0.001). Predicted probabilities demonstrated a progressive rise in odds of elevated UA lactate as Cat II % increases. When examining Cat II % across labor phases, each 1% increase during the latent phase was associated with a 0.6% increase in odds (aOR 1.006, 95% CI 1.003–1.008); this association strengthened with the addition of Cat II in the active phase (aOR 1.008, 95% CI 1.006–1.011), and further with inclusion of the second stage (aOR 1.009, 95% CI 1.007–1.012), suggesting a cumulative association across labor.

**Conclusion**: Higher Cat II % is independently associated with a small but significant increase in odds of elevated UA lactate. Findings across labor phases suggest that prolonged presence of Cat II may confer increasing risk.